# Supplementary material for: Knowledge, attitude and practice regarding diabetes and hypertension among school students of Nepal: A rural vs. urban study
Source: PLoS One. 2022 Aug 31;17(8):e0270186. doi: 10.1371/journal.pone.0270186 (PMC9432731; doi:10.1371/journal.pone.0270186)
Supplement: S1 File — (PDF) [file pone.0270186.s001.pdf]

KAP on Diabetes and Hypertension Questionnaire

A. Demographic Information

Age: ………………………………….

Sex: ☐ Male ☐ Female ☐ Others

Education (Grade of study): ……………………………..

Occupation of family head: ……………………………………

Qualification of family head: ………………………………………..

1. From where you get health related information? (You can choose multiple answers)

☐ School ☐ Television ☐ Internet ☐ Radio ☐ Newspaper

2. Does anyone in your family suffer from Diabetes?

☐ Yes ☐ No

3. Does anyone in your family suffer from Hypertension?

☐ Yes ☐ No

B. Knowledge, Attitude and Practice on Diabetes and Hypertension

4. Do you know what Diabetes is?
☐ Yes ☐ No ☐ Don't know

5. Is Diabetes a communicable disease?
☐ Yes ☐ No ☐ Don't know

6. . In your opinion, what are the causes of diabetes? (you can choose multiple options)

☐ Obesity ☐ Decreased Physical Activity ☐ Family history ofdiabetes
☐ Mental Stress

7. Do you know what complications Diabetes can cause to other organs?
   ☐ Yes ☐ No ☐ Don't know

8. If yes, which organs diabetes affect? (you can choose multiple options)
   ☐Eye disease ☐Kidney problems ☐Foot problems ☐Hypertension

   ☐Heart attack ☐Stroke

9. . Can Diabetes be prevented?
   ☐ Yes ☐ No ☐ Don't know

10. Diabetes can be prevented with dietary modification.

    ☐ Agree ☐ Disagree ☐ Neutral

11.. Regular exercise prevents Diabetes.

    ☐ Agree ☐ Disagree ☐ Neutral

12. If your family members or blood-related relatives have diabetes, you are also at risk of diabetes.

    ☐ Agree ☐ Disagree ☐ Neutral

13. Smoking worsens the complications of diabetes.

    ☐ Agree ☐ Disagree ☐ Neutral

14. Regular blood sugar monitoring helps to control Diabetes.

    ☐ Agree ☐ Disagree ☐ Neutral

15. Do you know what Hypertension is?

    ☐ Yes ☐ No ☐ Don't know

16. What is normal blood pressure?

    ☐100/90 ☐120/80 ☐110/70 130/90

17. Is Hypertension a communicable disease?

    ☐ Yes ☐ No ☐ Don't know

18. What are the symptoms of Hypertension? (You can choose multiple options)

☐ Headache ☐ Blurred vision ☐ Dizziness ☐ Shortness of breath

19. What are the causes of Hypertension? (You can choose multiple options)

☐ Lack of physical activity ☐ Mental Stress ☐ Family history of hypertension ☐ High salt intake ☐ Obesity

20. We should reduce salt intake to prevent Hypertension.

☐ Agree ☐ Disagree ☐ Neutral

21. For preventing Hypertension, we should eat plenty of fruits and vegetables.

☐ Agree ☐ Disagree ☐ Neutral

22. Regular checking of blood pressure is important.

☐ Agree ☐ Disagree ☐ Neutral

23. Smoking leads to Hypertension.

☐ Agree ☐ Disagree ☐ Neutral

24. Doing exercise regularly prevents Hypertension.

☐ Agree ☐ Disagree ☐ Neutral

25. How often do you add salt (or *Bire noon*) to your food right before you eat it or as you are eating it?

☐ Always ☐ Often ☐ Sometimes ☐ Rarely ☐ Never ☐ Don't know

26. How much salt do you think you consume?

☐ Far too much ☐ Too much ☐ Just the right amount ☐ Too little ☐ Far too little ☐ Don't know

27. i. In a typical week, on how many days do you eat fruit?

………………………….. (If zero days, go to 28)

ii. How many servings of fruit do you eat on one of those days?

……………………………

28. i. In a typical week, on how many days do you eat vegetables?

…………………………(If zero, go to 29)

ii.How many servings of vegetables do you eat on one of these days?

…………………………….

29. i. In a typical weeks, on how many days do you do vigorous intensity activities (activities that cause large increase in breathing or heart rate)?

……………………………. (If zero, go to C)

ii. How much time do you spend doing vigorous intensity activities on that typical day?

…………………………..
