# Supplementary material for: Knowledge, attitude and practice regarding diabetes and hypertension among school students of Nepal: A rural vs. urban study
Source: PLoS One. 2022 Aug 31;17(8):e0270186. doi: 10.1371/journal.pone.0270186 (PMC9432731; doi:10.1371/journal.pone.0270186)
Supplement: S2 File — (PDF) [file pone.0270186.s002.pdf]

ज्ञान, मनोवृत्ति र आचरण सर्वेक्षण
(मधुमेह र उच्च रक्तचाप)
प्रश्नावली

वैयक्तिक विवरण

उमेर ....

लिङ्ग ☐ पुरुष ☐ महिला ☐ अन्य

अध्ययनरत कक्षा .... पारिवारिक पेशा ....

अभिभावकको शैक्षिक योग्यता ....

१. तपाईंले स्वास्थ्य सम्बन्धी जानकारी कहाँबाट पाउनुहुन्छ? (एकभन्दा बढी उत्तरमा ठीक लगाउन सक्नुहुन्छ)

☐ विद्यालय ☐ टेलिभिजन ☐ इन्टरनेट ☐ रेडियो ☐ पत्रपत्रिका

२. के तपाईंको परिवारमा कसैलाई मधुमेह (सुगर रोग वा Diabetes) को समस्या देखिएको छ?

☐ छ ☐ छैन ☐ थाहा छैन

३. के तपाईंको परिवारमा कसैलाई उच्च रक्तचापको समस्या देखिएको छ?

☐ छ ☐ छैन ☐ थाहा छैन

४. तपाईंलाई मधुमेह भनेको के हो थाहा छ?

☐ छ ☐ छैन

५. के मधुमेह सरुवा रोग हो?

☐ हो ☐ होइन ☐ थाहा छैन

६. तपाईंको विचारमा मधुमेह के कारणले लाग्न सक्छ? (एकभन्दा बढी उत्तर रोज्न सक्नुहुन्छ)

☐ मोटोपना ☐ शारीरिक कसरतको कमी ☐ वंशाणुगत ☐ मानसिक तनाव ☐ अस्वस्थ खानपान

७. मधुमेहले शरीरका अन्य अंगहरुमा निम्त्याउन सक्ने जटिलताको बारेमा कत्तिको जानकार हुनुहुन्छ?

☐ छु ☐ छैन

८. यदि हुनुहुन्छ भने, यसले कुन कुन अंगहरुलाई असर गर्छ?

☐ आँखा ☐ मृगौला ☐ खुट्टामा समस्या ☐ उच्च रक्तचाप ☐ हृदयघात ☐ मष्तिष्क घात

९. के मधुमेहलाई रोकथाम गर्न सकिन्छ?

☐ सकिन्छ ☐ सकिदैंन ☐ थाहा छैन

१०. खानपिनमा उचित ध्यान दिन सकेमा मधुमेह रोकथाम गर्न सकिन्छ।

☐ सहमत ☐ असहमत ☐ न त सहमत न असहमत

११. नियमित रुपमा शारीरिक व्यायाम गरेमा मधुमेहको जोखिम कम हुन्छ।

☐ सहमत ☐ असहमत ☐ न त सहमत न असहमत

१२. यदि तपाईंको परिवारमा कसैलाई मधुमेह भएको छ भने, तपाईंलाई पनि यो रोग लाग्न सक्छ।

☐ सहमत ☐ असहमत ☐ न त सहमत न असहमत

१३. धुम्रपानको कारणले मधुमेहका समस्याहरु अझै बढ्ने गर्छन्।

☐ सहमत ☐ असहमत ☐ न त सहमत न असहमत

१४. नियमित रुपमा रगतमा सुगर (चिनि) को मात्रा जाँच गरेमा मधुमेह नियन्त्रण गर्न सकिन्छ।

☐ सहमत ☐ असहमत ☐ न त सहमत न असहमत

१५. के तपाईंलाई उच्च रक्तचाप भनेको के हो थाहा छ?

☐ छ ☐ छैन

१६. मानिसको सामान्य रक्तचाप (ब्लड प्रेसर) कति हुन्छ ?

☐ १००/९० ☐ १२०/८० ☐ ११०/७० ☐ १३०/९०

१७. के उच्च रक्तचाप सरुवा रोग हो?

☐ हो ☐ होइन ☐ थाहा छैन

१८. उच्च रक्तचापको लक्षणहरु के के हुन्? (एकभन्दा बढी उत्तरहरु रोज्न सक्नुहुन्छ)

☐ टाउको दुख्ने ☐ आँखा धमिलो देख्ने ☐ रिँगटा लाग्ने ☐ श्वास फेर्न गाह्रो हुने

१९. उच्च रक्तचाप कुन कुन कारणले हुन सक्छ? (एकभन्दा बढी उत्तरहरु रोज्न सक्नुहुन्छ)

☐ शारीरिक व्यायामको कमीले ☐ मानसिक तनावले ☐ वंशाणुगत ☐ धेरै नुन खानाले ☐ मोटोपनले

२०. उच्च रक्तचाप रोकथाम गर्नको लागि हामीले खाने नुनको मात्रा कम गर्नुपर्छ।

☐ सहमत ☐ असहमत ☐ न त सहमत न असहमत

२१. उच्च रक्तचाप रोकथामका लागि हामीले प्रशस्त फलफुल र सागसब्जी खानुपर्छ।

☐ सहमत ☐ असहमत ☐ न त सहमत न असहमत

२२. नियमित रुपमा रक्तचाप (ब्लड प्रेसर) को जाँच गर्नु महत्वपूर्ण छ।

☐ सहमत ☐ असहमत ☐ न त सहमत न असहमत

२३. धुम्रपान गरेमा उच्च रक्तचाप हुन सक्छ।

☐ सहमत ☐ असहमत ☐ न त सहमत न असहमत

२४. नियमित व्यायाम गर्नाले उच्च रक्तचापको जोखिमबाट बाँच्न सकिन्छ।

☐ सहमत ☐ असहमत ☐ न त सहमत न असहमत

२५. तपाईं आफुले खाना खानु अघि वा खाँदै गर्दा कत्तिको नुन थपेर खाने गर्नुहुन्छ?

☐ सधैं ☐ धेरै जसो ☐ कहिलेकाहीँ ☐ विरलै ☐ कहिल्यै ☐ थप्दिन थाहा छैन

२६. तपाईंको विचारमा तपाईंले कति मात्रामा नुन खानुहन्छ?

☐ एकदमै धेरै ☐ धेरै ☐ सही मात्रामा ☐ कम ☐ एकदमै कम ☐ थाहा छैन

२७. (क) सामान्यतया, तपाइँ एक हप्तामा कति दिन फलफुल खानुहुन्छ?

....

(ख) यदि खानुहुन्छ भने, एक दिनमा कति चोटि खानुहुन्छ?

....

२८. (क) सामान्यतया, तपाइँ एक हप्तामा कति दिन सागसब्जी खानुहुन्छ?

....

)ख) यदि खानुहुन्छ भने, एक दिनमा कति चोटि खानुहुन्छ?

....

२९. (क) सामान्यतया, तपाइँ एक हप्तामा कति दिन पसिना आउने गरी शारीरिक क्रियाकलाप गर्नुहुन्छ? (तपाईंको धड्कन वा श्वास प्रश्वासको गति बढ्ने गरी)

....

(ख) यदि गर्नुहुन्छ भने, एक दिनमा कति समय उक्त क्रियाकलापमा बिताउनु हुन्छ?

....
