# Supplementary material for: Knowledge, attitude and practice regarding diabetes and hypertension among school students of Nepal: A rural vs. urban study
Source: PLoS One. 2022 Aug 31;17(8):e0270186. doi: 10.1371/journal.pone.0270186 (PMC9432731; doi:10.1371/journal.pone.0270186)
Supplement: Attachment — Submitted filename: Response to Reviewers.docx [file pone.0270186.s004.docx]

Answer to comments of reviewer 1:

Thank you for the comments.

1. **General: a language and grammar edit are essential on this manuscript due to many errors through the text. Also, the citations are doubled and need revision.**

- Necessary grammatical edits have been done after revision. We have rechecked the citations and we didn't find any duplications. However, some authors are common in more than one reference as they have published series of articles on NCDs in Nepal.

2. **Title: since this study evaluates KAP regarding only diabetes and hypertension, authors may replace the term “selected NCDs” with the two mentioned conditions to present a clear title of study.**

- In the title, "selected NCDs" has been replaced with "diabetes and hypertension".

3. **Abstract: although this part summarizes the manuscript very well, the prepared abstract is too long and should be briefer for a faster review of the manuscript. Therefore, it is suggested that authors drop unnecessary details of the abstract.**

- Some parts of the abstract which are less necessary have been dropped.

4. **Introduction: the provided statistics of the NCDs’ burden is outdated and authors may use more updated and recent estimations and studies to introduce the importance of NCDs. One of the robust studies in this regard is the Global Burden of Disease Study which its recent iteration known as GBD 2019 provides the most statistics.**

- The data used were from latest WHO country profile 2018. Newest data on NCDs from GBD 2019 have been added in introduction section.

5. **Methods, Study Settings and Participants: the selected three type of study settings is misleading. It is highly suggested that authors select only two major population of urban versus rural students to understand the difference easier and also to benefit the health policy makers.**

- We discussed with our statistician and supervisors regarding the adjustment in the type of study settings. The study settings in this study represents three levels of administrative divisions of Nepal, the major basis of which is level of urbanization. So, they have advised that these settings represent the status of KAP in different local levels of the country. These findings will be beneficial in policy making as well as health program designing, as local government has an important role in designing and conducting the health programs in local levels.

6. **Methods, Study Tools: the referred previous studies need proper citations. Also, the STEPS survey should be elaborated for the audience who are not familiar with this study.**

- The referred previous studies have been cited.
- A description of STEPS survey has been added in this section.

7. **Methods, Data analysis: all recruited criteria from WHO methods in this study should be clearly explained.**

- Only few questions on physical activity, vegetable and fruit consumption and salt intake have been taken from STEPS survey to include in the practice section of the KAP questionnaire. They have been presented as mean values, frequency and percentage as per the WHO STEPS survey. This has been mentioned in the data analysis section.

8. **Results: since this section presents a huge number of results, it is highly suggested that authors make a revision on this section and rearrange them in 3-4 main subsections to make following the results easier for the audience. Also, the provided tables and figures are appropriate and authors may delete some of the text and refer the reader to the tables and summarize the section.**

- The three sub-sections "knowledge scores", "attitude scores" and "level of knowledge and attitude" have been merged into one- "knowledge and attitude of students on diabetes and hypertension".
- The results section has been rearranged in 5 main sub-sections- "Socio-demographic characteristics", "sources of information on diabetes and hypertension", "knowledge and attitude of students on diabetes and hypertension", "Practice behaviors on diabetes and hypertension" and "Effects of socio-demographic factors on knowledge and attitude on diabetes and hypertension.

9. **Discussion: it is suggested that authors begin this section with a general interpretation of the findings instead of providing numbers of the results section**

- The numerical figures have been removed from the discussion section and only the general interpretation of the findings has been mentioned.

10. **Discussion: one paragraph is needed in this section discussing the ongoing programs regarding the control of NCDs in Nepal and discussing its limitations and the gaps, since it is necessary when authors investigate the KAP regarding NCDs in this manuscript.**

- A paragraph explaining about ongoing programs regarding the control of NCDs in Nepal has been added in the discussion section.

**Answers to comments of reviewer 2:**

Thank you for the feedback.

**2. Inclusion/exclusion criteria should be more detailed.**

- Consecutive sampling was used and all the students of class 9 and 10 of the selected schools were included in the study. Only the students who were absent at the time of data collection were excluded from the study. There were no other exclusion criteria.

**References:**

**References: The manuscript employed the use of Harvard style referencing but requires editing to correct some errors noticed e.g., Listing of references: Shouldn’t this be in alphabetical order? Shouldn’t the journal name be italics? Shouldn’t the list of authors that are more than 5 be reflected as et al?
I suggest the authors should revise Harvard referencing style and make necessary corrections.**

- Vancouver style referencing has been used in the manuscript as per the journal submission guideline.
- The references have been listed numerically as per their citation in the manuscript.
- It has been stated that italics may not be required in Vancouver style.
- In Vancouver, the list of authors that are more than 6 are reflected as et al. This has been used in the manuscript.

Journal Requirements:

1. **Please ensure that your manuscript meets PLOS ONE's style requirements, including those for file naming.**

- PLOS ONE's style requirements have been checked and the manuscript has been adjusted accordingly.

2. **You indicated that you had ethical approval for your study. In your Methods section, please ensure you have also stated whether you obtained consent from parents or guardians of the minors included in the study or whether the research ethics committee or IRB specifically waived the need for their consent.**

- Mention of parental consent has been added in the method section.
